# 

**Published:** 1896-03-21

**Authors:** 


### CONTENTS.

**The Presidency of the Royal College of Physicians** - 407
**The Old and the New Vitalism** - - - - - - 408
**Medical Progress and Hospital Clinics—**
On the Treatment of Femoral Hernia. By W. McAdam Eccles, M.S.Lond. I.—Reducible Femoral Hernia - - - 413
Progress in Medicine.—Diseases of the Nervous System (*continued*) - - - - - - - - - - - 414
Diseases of the Intestines - - - - - - - - - - 416
Progress in Surgery.—Surgery of the Liver and Gall Bladder - - - - - - - - - - - - - 417
New Appliances and Things Medical - - - - - 418
**Editor's Letter-Box** - - - - - - - - - - 418
**The Institutional Workshop—**
Hospital Expenditure: The Commissariat. XII.—The Cost of What Patients Provide - - - - - - - - - - 419
Hospital Meetings - - - - - - - - - - - - 419
**Dyspepsia and Consumption.** By Solomon C. Smith, M.D. III.—Treatment - - - - - - - - - - - - 409
**Modern Sociology.**—Lunacy and Pauperism - - - - 410
**Annotations.**—The Notification of Measles—"Medical Temperance"—Public-houses and Sanitary Authorities - - - - 411
**Notes and News** - - - - - - - - - - - - 412

### EXTRA SUPPLEMENT.—THE NURSING MIRROR.

**News from the Nursing World.**—Royalties Among the Hospitals—Royal Hospital, Richmond—Hampshire Nurses' Institute—The Male Nurse in America—Middlesbrough Nursing Association—Macclesfield General Infirmary—Another Workhouse "Accident"—Liverpool Eye and Ear Hospital—Lectures at Caterham—Private Nursing Abroad—Short Items ccxvii

**The National Hospital for Paralysis and its Lady Superintendent** - - - - - - - - - - - ccxx

**Training School Registries.**—II. - - - - - - ccxxi

**The Nurses' Wedding Present to H.R.H. Princess Maud** - - - - - - - - - - - - - ccxix

**The Royal National Pension Fund for Nurses** - - ccxxii

**Our American Letter** - - - - - - - - - ccxxiv

**East-end Mothers' Home** - - - - - - - - ccxxv

**Everybody's Opinion** - - - - - - - - - ccxxv

**District Nursing in Ireland** - - - - - - - ccxxv

**Notes and Queries** - - - - - - - - - - ccxxvi

**For Reading to the Sick** - - - - - - - - ccxxvi

